# Dynamic diffraction artefacts in Bragg coherent diffractive imaging

**DOI:** 10.1107/S1600576718000274

**Published:** 2018-02-01

**Authors:** Wen Hu, Xiaojing Huang, Hanfei Yan

**Affiliations:** a National Synchrotron Light Source II, Brookhaven National Laboratory, PO Box 5000, Upton, New York 11973, USA

**Keywords:** dynamic diffraction, Bragg coherent diffractive imaging, iterative phase reconstruction

## Abstract

This article reports a theoretical study on the reconstruction artefacts in Bragg coherent diffractive imaging caused by dynamical diffraction effects. It is shown that, unlike the absorption and refraction effects that can be corrected after reconstruction, dynamical diffraction effects have profound impacts on both the amplitude and the phase of the reconstructed complex object, causing strong artefacts. At the dynamical diffraction limit, the reconstructed shape is no longer correct, as a result of the strong extinction effect. Simulations for hemispherical particles of different sizes show the type, magnitude and extent of the dynamical diffraction artefacts, as well as the conditions under which they are negligible.

## Abstract 2

The article presents a theoretical study on dynamic artefacts in the reconstruction of Bragg coherent diffractive imaging using an iterative phase retrieval algorithm.

## 1. Introduction   

Bragg coherent diffractive imaging (BCDI) emerged recently as a powerful tool to probe the internal deformation field of a nanocrystal on the basis of coherently diffracted X-ray data (Robinson *et al.*, 2001 ▸; Pfeifer *et al.*, 2006 ▸). It has attracted a great deal of attention and interest owing to its unique capability for quantitative three-dimensional strain mapping (Clark *et al.*, 2003 ▸; Huang *et al.*, 2015 ▸; Ihli *et al.*, 2016 ▸; Ulvestad *et al.*, 2015 ▸, 2017 ▸). In this technique, a sequence of two-dimensional diffraction patterns are recorded at rocking angles near a Bragg peak and then assembled into a three-dimensional reciprocal space map of the crystal structure. Although only intensities in reciprocal space are measured, the unknown complex object function in real space can be reconstructed by phase-retrieval algorithms (Fienup, 1982 ▸; Miao *et al.*, 1998 ▸; Williams *et al.*, 2003 ▸). In the Bragg case, the amplitude of the reconstructed function corresponds to the Bragg electron density, while its phase represents the internal displacement field. Owing to the brilliance and coherence of the source at third-generation synchrotron facilities, and the development of phase-retrieval algorithms, many successful BCDI applications have been reported (Miao *et al.*, 2003 ▸; Song *et al.*, 2007 ▸; Yang *et al.*, 2013 ▸; Robinson & Harder, 2009 ▸; Ihli *et al.*, 2016 ▸; Ulvestad *et al.*, 2017 ▸) and have demonstrated the great capability of BCDI in measuring strain in three dimensions at the nanoscale.

Current applications, however, are limited to nanocrystals with sizes ranging from ∼100 to ∼700 nm. The obstacle of going smaller is mostly the coherent flux. The diffraction signal from smaller nanocrystals has poor signal-to-noise ratio. The difficulty of going larger is twofold. On the one hand, satisfying the oversampling condition for larger crystals requires a long detector-to-sample distance. In some cases it becomes practically prohibitive. On the other hand, the wavefield inside a nearly perfect large crystal undergoes a multiple-scattering process, a phenomenon known as the dynamical diffraction effect. The iterative phase-retrieval algorithm used in BCDI is based on the assumption that X-rays diffract kinematically from a nanocrystal, in which case the assembled diffraction patterns correspond to a reciprocal space map and can be calculated by a three-dimensional Fourier transform of the Bragg electron density and the displacement field of the nanocrystal. The phase-retrieval algorithm propagates a complex quantity back and forth between reciprocal and real space using a Fourier transform. When dynamical diffraction occurs, this mapping relationship can be broken. Consequently, BCDI may lead to erroneous results with strong artefacts. In the literature, dynamical diffraction induced phase variation in the transmitted beam was investigated before (Gorobtsov & Vartanyants, 2016 ▸), but there has not been a thorough discussion on the reconstruction artefacts due to dynamical diffraction effects.

In this work, we present a theoretical study on this topic, with the aim of identifying dynamical diffraction artefacts in the retrieved amplitude and phase. It is important to understand the type, extent and magnitude of these dynamical artefacts in the reconstruction so that they are not interpreted as real structural variations. It is also important to explore the range of validity of kinematical diffraction so that experimental conditions can be optimized to minimize the dynamical diffraction effect. We first discuss the formulism difference in kinematical and dynamical diffraction. We then compare the difference in far-field diffraction patterns calculated using different models, and discuss the dynamical diffraction artefacts in BCDI by comparing the reconstruction results with those obtained from kinematical diffraction calculation under the same conditions. A systematic study is carried out to investigate the dependence of dynamical artefacts on crystal size. It is shown that a strong extinction effect can lead to an incorrect crystal shape in the reconstructed amplitude, and the retrieved phase can exhibit a complex pattern that is not related to the true displacement field. We show that dynamical diffraction artefacts are negligible for a crystal size below the extinction depth, but when its size approaches the *Pendellösung* distance, even the shape cannot be reconstructed correctly. As a result, energy and reflection indices that ensure this criterion is satisfied should be chosen to minimize these undesirable artefacts in a real BCDI experiment.

## 2. Kinematical and dynamical diffraction formulism   

**Figure 1:**
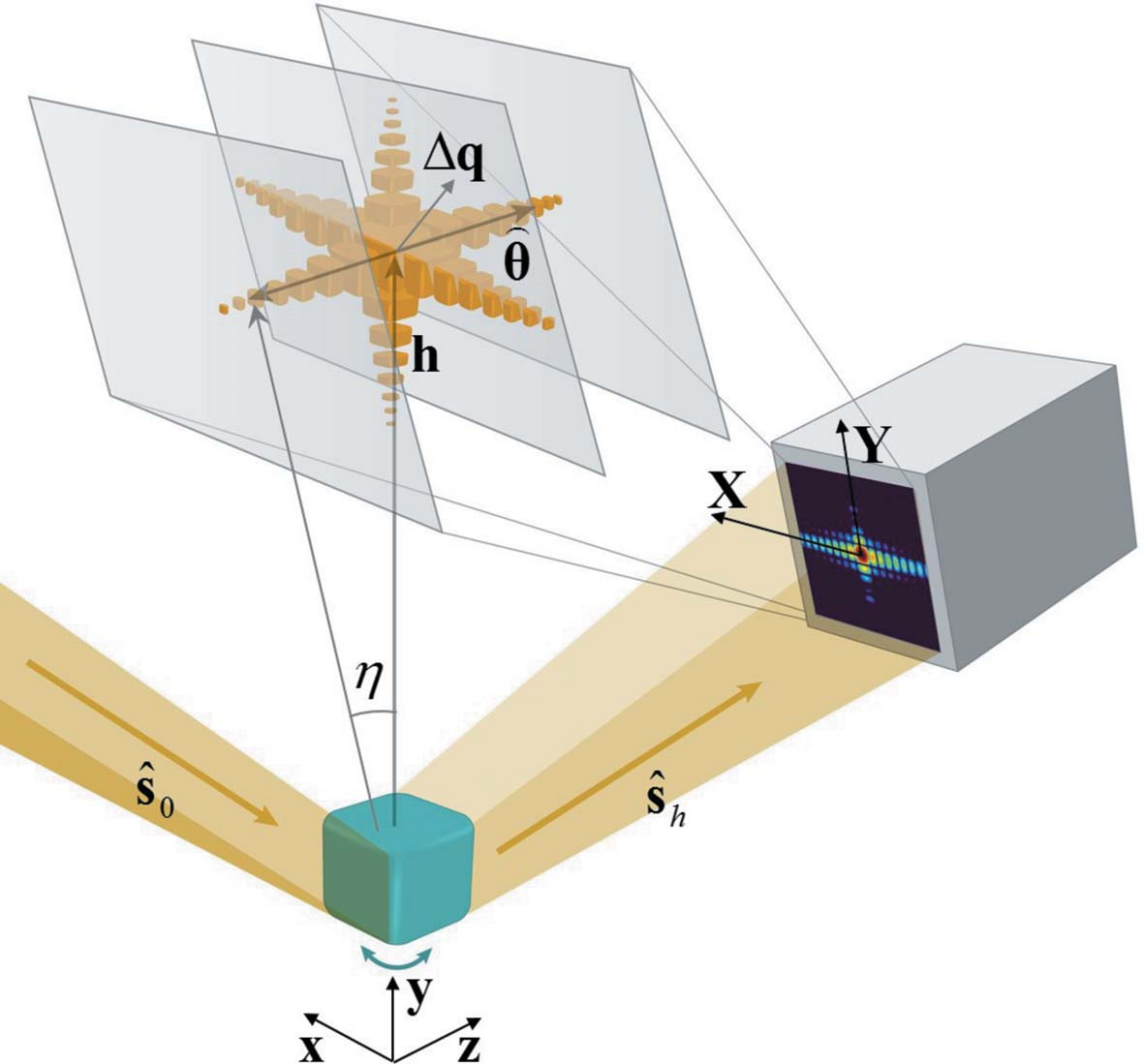
A schematic drawing of the diffraction geometry. X-rays are diffracted by a crystal and then captured by a pixel-array detector placed perpendicular to the diffracted beam. The recorded diffraction pattern corresponds to a slice in the reciprocal space near the reciprocal lattice vector, **h**, of the excited reflection. At each rocking angle, this slice is shifted along the swing direction of the reciprocal lattice vector, 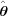. An assembly of far-field diffraction patterns at different angles forms a data set describing the intensity distribution in the reciprocal space in three dimensions.

Kinematical diffraction theory is an approximation to the more rigorous dynamical diffraction theory and is valid for a small or ideally imperfect crystal where the diffracted wave is very weak. In such cases, we can only consider the interactions between the primary incident wave and the atoms, and neglect all high-order effects (Born approximation). If we further ignore photoelectric absorption and the small difference from unity of the refractive index in a medium that causes a phase change, the recorded diffraction patterns on a far-field two-dimensional detector at a set of crystal rocking angles can be expressed for an incident plane wave as (Warren, 1990 ▸; Vartanyants & Robinson, 2001 ▸)
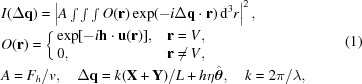
where 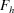 is the structure factor representing scattering from a unit cell, *v* is the volume of the unit cell, *V* is the volume of the crystal, *L* is the distance from the sample to detector, **X** and **Y** are position vectors in the detector plane, η is the angular deviation from the exact Bragg angle, 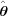 is an angular unit vector in the rotation plane, λ is the wavelength in vacuum, **h** is the unstrained reciprocal lattice vector, and **u** is the displacement vector. Their relationships are displayed in Fig. 1 ▸. Here we assume the structure factor is a constant across the crystal so that it is taken out of the integral. As a matter of fact, the right-hand side of equation (1) is the square modulus of a three-dimensional Fourier transform. Given that the oversampling condition is satisfied, it has been shown that the complex object function, *O*, can be reconstructed from the square modulus of its Fourier transform using iterative phase-retrieval algorithms (Fienup, 1982 ▸; Miao *et al.*, 1998 ▸; Elser, 2003 ▸; Williams *et al.*, 2003 ▸; Marchesini, 2007 ▸). From its phase, we can obtain the strain information. As can been seen, three-dimensional Fourier transformation is the mathematical basis of BCDI.

In the case of dynamical diffraction, however, the diffracted wave is too strong to be negligible. Consequently, the multi-wave scattering effect, which takes into account interactions between the diffracted wave and the atoms, needs to be considered as well. X-ray dynamical diffraction theory has been developed for a long time and there are many monographs (Pinsker, 1978 ▸; Authier, 2001 ▸). A dynamical diffraction calculation from crystallites with an arbitrary shape, however, remains a challenging problem because of the mixture of boundary conditions in Bragg and Laue geometry. In a recent publication, Yan & Li (2014 ▸) developed a modelling approach based on the integral representation of the Takagi–Taupin equations (Takagi, 1962 ▸; Taupin, 1964 ▸) that unifies both boundary conditions. It allows a rigorous dynamical diffraction calculation from a crystallite with arbitrary shape. The iterative solving process represents the transition from kinematical to dynamical diffraction, which is a great advantage compared to other methods. Throughout this work, we use this approach to calculate both kinematical and dynamical diffraction patterns. In their paper, Yan and Li derived an analytical expression of the far-field diffraction pattern:
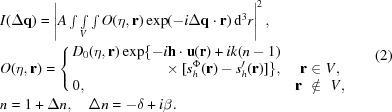
Both δ and β are very small quantities. The former results in a phase change and the latter determines the absorption. *D*_0_ is the envelope function of the transmitted wave, *n* is the complex refractive index of the crystal and 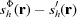 is the path difference along the diffracted beam direction, which accounts for the refraction and photoelectric absorption effects of the diffracted beam associated with the refractive index deviation from unity. Note that similar effects of the transmitted beam are included in the envelope function of the transmitted wave, *D*_0_, though it is not expressed explicitly (Yan & Li, 2014 ▸). Compared to the expression in equation (1), the object function now includes two more terms. If the transmitted wave is independent of the rocking angle of the crystal, equation (2) indicates that a Fourier transform assumption is still valid, with the consideration of refraction and photoelectric absorption along the transmitted and diffracted path inside the crystal. The difference, as compared to equation (1), is that a correction accounting for the non-unity of refractive index is needed to retrieve the correct displacement function, **u**. When dynamical diffraction occurs, however, *D*_0_ is also a strong function of the rocking angle. In other words, it depends on Δ**q**. As a result, it is not a Fourier transform and the wavefield in the detector plane does not represent a slice cut on the Ewald sphere of the crystal.

We emphasize that the propagation of the diffracted wave from the crystal to the detector is always governed by a two-dimensional Fourier transform as long as the Fraunhoffer diffraction condition is satisfied, and it is independent of the diffraction mode in the crystal. One should not confuse it with equation (2), which accounts for both Bragg diffraction inside the crystal and wave propagation to the detector. Under the kinematical diffraction condition, equation (2) states that the assembled far-field diffraction patterns near the Bragg angle can be interpreted as a three-dimensional Fourier transform of the crystal function, with its amplitude representing the absorption effect and its phase reflecting the change of the refractive index and the displacement field in the crystal. When dynamical diffraction occurs, this relationship is broken.

## 3. Difference in far-field diffraction patterns   

**Figure 2:**
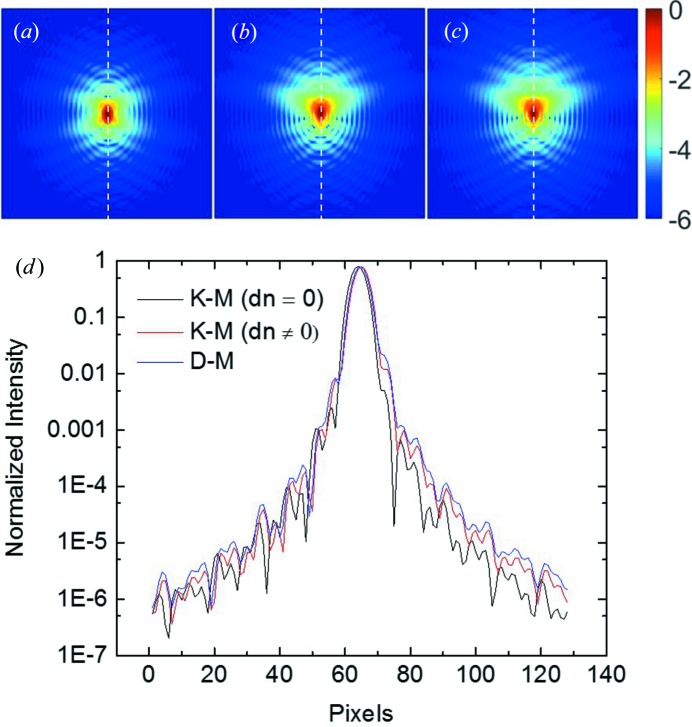
Far-field diffraction patterns in logarithmic scale for an Au hemispherical crystal (500 nm radius) at the Bragg angle, calculated using the kinematical diffraction model without (*a*) and with (*b*) the consideration of absorption/refraction effects, and the dynamical diffraction model (*c*). The energy is 7.5 keV and the reflection index is 002. (*d*) shows line intensity variations across the centre of these images (white dashed lines) in logarithmic scale.

We first studied the difference in far-field diffraction patterns calculated using two models. We considered a hemispherical Au particle with a radius of 500 nm. Au 002 Bragg diffraction was selected. We assumed that a two-dimensional detector placed perpendicular to the diffracted beam intercepts the beam and records the diffraction pattern at a distance of 1 m. The detector has 128 × 128 pixels and a pixel size of 55 µm. The incident beam was considered as a plane wave at 7.5 keV. At the exact Bragg angle (η = 0), far-field diffraction patterns were calculated using a kinematical diffraction model (K-M, Δ*n* = 0), a kinematical diffraction model with the consideration of absorption and refraction effects (K-M, Δ*n* 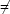 0), and a dynamical diffraction model (D-M). For simplicity, the particle was assumed to be a perfect single crystal with no strain, *i.e.* **u** = **0**. Figs. 2 ▸(*a*)–2 ▸(*c*) show the results. In reciprocal space, the images correspond to a slice of the diffraction intensity distribution at a tilted angle with respect to the reciprocal lattice vector, **h**, as shown in Fig. 1 ▸. One can see that these images all exhibit similar interference fringes associated with the shape of the particle. The latter two images are almost identical, and their diffraction patterns extend further into the high-*q* region. To quantify the difference, line profiles of the intensity along the vertical direction (as marked by the white dashed line) are depicted in Fig. 2 ▸(*d*). In logarithmic scale, an appreciable difference can be observed. We notice that the latter two models yield a diffraction peak shifted toward a higher angle. Recall that the refractive index of the crystal is slightly smaller than unity. As a result, the wavelength is larger in the crystal than in vacuum, and so is the Bragg angle. The non-unity refractive index also causes a slight change of the interference pattern. With the consideration of the refraction effect, the kinematical and dynamical models differ mostly in the relative intensities of the side lobes. In addition, the dynamical model has less visibility of the fringes. The question arises here as to how these subtle differences in the diffraction pattern will impact the reconstruction of the particle shape and strain field when using the phase-retrieval algorithm, and whether a correction can be applied afterwards.

## 4. Absorption/refraction effect *versus* dynamical diffraction effect   

For the same 500 nm radius Au hemisphere, we computed diffraction patterns at 128 angles with an angular step size of 0.0039° using the models mentioned in the preceding section. The angular step size was determined by matching the resolution in detector coordinates (Williams *et al.*, 2003 ▸). The oversampling ratio was estimated from the ratio of the size of the entire array (128 × 128 × 128) to the size of the support obtained from the shrink-wrap method in BCDI reconstruction. This sets an oversampling ratio of about 105 and is sufficient for a phase-retrieval reconstruction (Miao *et al.*, 1998 ▸; Marchesini, 2007 ▸). We then reconstructed the complex object function from the 128 × 128 × 128 diffraction data set. A shrink-wrap procedure was used to constrain the support gradually (Marchesini *et al.*, 2003 ▸). To rule out any numerical errors originating from the phase-retrieval algorithm itself, we used the same procedures and reconstruction parameters in all cases. A combination of hybrid-input-output and error-reduction algorithms were applied (Gerchberg & Saxton, 1972 ▸; Fienup, 1982 ▸; Pfeifer *et al.*, 2006 ▸), and the shrink-wrap procedure was triggered twice and applied to the support as a constraint with an interval of ten fixed-support iterations (Clark *et al.*, 2003 ▸; Ihli *et al.*, 2016 ▸).

**Figure 3:**
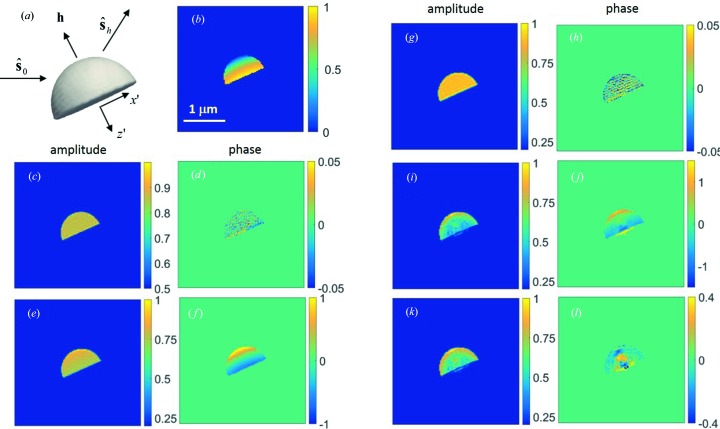
(*a*) Au hemispherical crystal (500 nm radius) reconstructed in three dimensions using the kinematical model and viewed along the axis perpendicular to the diffraction plane (defined by the incident and diffracted directions). The Au 002 reflection at 7.5 keV is assumed. (*b*) Optical path difference along the incident and diffracted beam directions inside the crystal, which determines the attenuation and the amount of phase shift the X-ray wave experiences as a result of a non-unity complex refractive index. (*c*) and (*d*) are the cross-sectional views of the reconstructed amplitude and phase in the central diffraction plane obtained from the K-M (Δ*n* = 0) data set, respectively. (*e*) and (*f*) are the reconstructed amplitude and phase in the same plane obtained from the K-M (Δ*n* 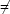 0) data set, and (*g*) and (*h*) are the corresponding images after absorption/refraction correction. (*i*) and (*j*) are the reconstructed amplitude and phase obtained from the D-M data set, and (*k*) and (*l*) are the corresponding images after absorption/refraction correction.

Fig. 3 ▸(*a*) shows the reconstructed three-dimensional object for the K-M (Δ*n* = 0) data set; the incident beam is along the 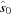 direction and the exit beam is along the 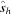 direction. For this study, we focused on the central diffraction plane which cuts through the centre of the hemisphere in Fig. 3 ▸(*a*). In the following discussion, we will only show the reconstructed results in this diffraction plane. The optical path of the beam at each position in the crystal was calculated and is shown in Fig. 3 ▸(*b*), including contributions from both incident and diffracted beams. For a radius of 500 nm and a Bragg angle of 23.9°, the optical path varies from zero at the top of the hemisphere to 1 µm at the bottom surface. Fig. 3 ▸(*c*) and 3 ▸(*d*) are the reconstructed amplitude and phase in the central diffraction plane. One can see nearly constant amplitude and phase with an r.m.s. error of 0.03 and 0.02, respectively. These small fluctuations are numerical errors originating from the adopted reconstruction algorithm at the given oversampling ratio and are considered to be intrinsic. Any larger variations are artefacts due to other factors.

As we discussed before, the non-unity refractive index will result in a complex function that can be calculated from the optical path difference inside the crystal. The optical path shown in Fig. 3 ▸(*b*) corresponds to a phase change as large as −2 radians and needs to be corrected. The reconstruction amplitude and phase obtained from the K-M (Δ*n* 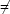 0) data set are plotted in Figs. 3 ▸(*e*) and 3 ▸(*f*), and absorption/refraction-corrected images are shown in Figs. 3 ▸(*g*) and 3 ▸(*h*). The correction works as expected and completely removes artefacts induced by refraction and absorption effects. The r.m.s. errors of the corrected amplitude and phase were 0.03 and 0.03, respectively. The corrected images of Figs. 3 ▸(*g*) and 3 ▸(*h*) barely show any difference from Figs. 3 ▸(*c*) and 3 ▸(*d*). This is not a surprising result since equation (2) can still be considered as a three-dimensional Fourier transform even with the absorption and refraction taken into account. We point out that the refraction effect causes a shift of the peak position (Fig. 2 ▸ *d*), which resulted in a linear phase ramp in the reconstruction (a property of Fourier transform). In our case we do not remove this phase ramp at the end of the reconstruction since it corresponds to the refraction effect.

**Figure 4:**
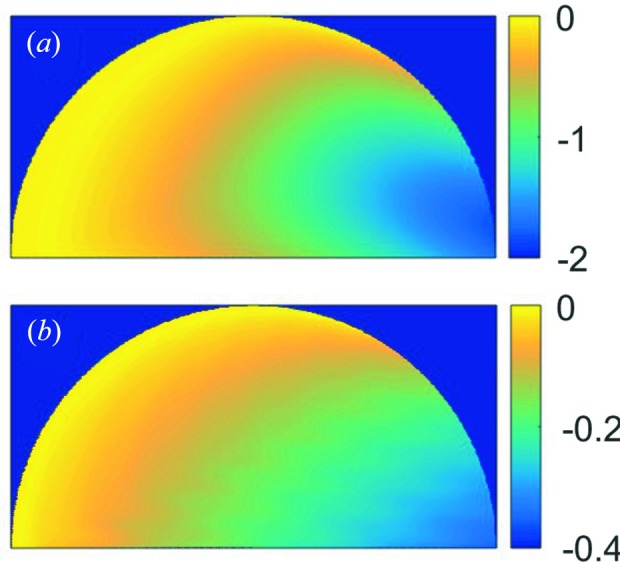
Transmitted wave intensity variations inside the Au hemispherical crystal (500 nm radius) at the Bragg angle (*a*) and −0.1° away (*b*). Intensity is in logarithmic scale. The attenuation coefficient in the former case is five times that in the latter because of the extinction effect.

The D-M data set leads to very different results. Figs. 3 ▸(*i*) and 3 ▸(*j*) are the reconstructed amplitude and phase, and Figs. 3 ▸(*k*) and 3 ▸(*l*) are the images with absorption/refraction correction. First, we observe that the convergence of the reconstruction is slower and the residual error is larger when using the D-M data set. This indicates an inconsistency of the dynamical diffraction model with the phase-retrieval algorithm, even though the data are perfect and all parameters used in the reconstruction are the same. Bear in mind that only under a kinematical diffraction condition does the far-field diffraction image correspond to a slice on the Ewald sphere of the crystal. Second, we observe that absorption and refraction corrections cannot remove all the observed variations. The object after correction shows a core–shell-like structure. The core has a weaker amplitude. The phase varies significantly from the shell to the core. The reconstructed amplitude and phase are not as smooth as those in the first two cases, with r.m.s. fluctuations of 0.12 and 0.06. Right under the bottom surface, we start to see a small phantom crystal that should not exist. As we can see from this comparison, the subtle changes of the diffraction pattern from the kinematical to dynamical models depicted in Fig. 2 ▸(*d*) can have a profound impact on the reconstruction. From equation (2) we see the root cause is that the envelope function of the transmitted wave is a function of the rocking angle; therefore the assembly of the diffracted waves arriving at the detector plane can no longer be formulated into a three-dimensional Fourier transform of the crystal. Even if the phase of the diffracted wave is known, a direct inverse three-dimensional Fourier transform will not correctly recover the object function, *O*. A recent paper showed similar artefacts when an inverse Fourier transform was directly applied to the simulated complex wavefield in the detector plane (Shabalin *et al.*, 2017 ▸). For a better understanding of this issue, in Figs. 4 ▸(*a*) and 4 ▸(*b*) we plot the transmitted wave intensity distribution inside the crystal at two angles. One angle is far away from the Bragg angle so it mostly suffers from photoelectric absorption, and the other is at the Bragg angle so extinction is strongly excited. The transmitted intensity decreases much faster in the latter case, and therefore a correction based on a constant refraction index fails.

## 5. Size dependence of dynamical diffraction effects   

**Figure 5:**
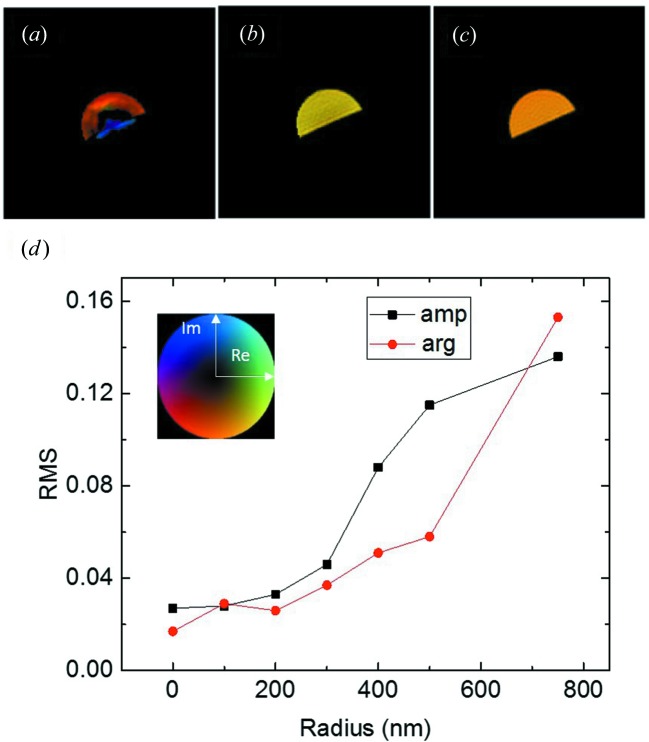
Reconstructed complex object function in the central diffraction plane of particles with radii of 750 nm (*a*), 300 nm (*b*) and 100 nm (*c*). The brightness and colour of the image represent the amplitude and phase of the complex function [see the inset image in (*d*)]. All the results have been corrected for the absorption and refraction effects. (*d*) shows the r.m.s. fluctuations of the reconstructed amplitude and phase in the central diffraction plane as a function of the size. The size zero refers to the kinematical diffraction limit.

Currently there is no good way to remove dynamical diffraction artefacts in the BCDI reconstruction without the development of an advanced phase-retrieval algorithm involving a dynamical diffraction calculation. Therefore, it is important to know the conditions under which the artefacts are negligible. As we discussed in the preceding section, whether or not a kinematical approximation is valid boils down to the dependence of the envelope function of the transmitted wave, *D*_0_, on the rocking angle. If it is nearly unchanged at different angles, one would expect very minimal dynamical diffraction effects. To ensure this condition, the size of the particle has to be smaller than the extinction depth defined as
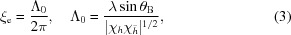
where 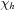, 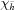 are the Fourier components of the dielectric susceptibility and Λ_0_ is called the *Pendellösung* distance in Laue geometry (or extinction distance in Bragg geometry) (Authier, 2001 ▸). For a particle reaching the size of Λ_0_ and beyond, we are at the dynamical diffraction limit. For simplicity, in equation (3) a symmetric reflection case is assumed. For the Au 002 reflection at 7.5 keV, the extinction depth, 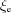, is 126 nm, and the *Pendellösung* distance, Λ_0_, is 792 nm. We expect that the dynamical diffraction artefacts will diminish as the size decreases and eventually vanish. To illustrate this tendency, in Figs. 5 ▸(*a*)–5 ▸(*c*) we show reconstruction results for hemispheres with radii of 750, 300 and 100 nm, respectively. A recent study reveals that, within a kinematic diffraction regime, the image reconstruction quality is subject to measurement parameters, such as oversampling conditions and detection dynamic range (Öztürk *et al.*, 2017 ▸). To ensure a fair comparison, we scaled the detector-to-sample distance and the rocking angle so that the oversampling ratio was the same. At a radius of 750 nm where dynamical diffraction is dominant (Fig. 5 ▸ *a*), even the reconstructed shape is incorrect. It shows a thin shell and a phantom crystal at the bottom, with a gap in between. The phase variation is over π/2. At a radius of 300 nm (Fig. 5 ▸ *b*), reconstruction artefacts were visible, but may be still manageable. At a radius of 100 nm, which is below the extinction depth (Fig. 5 ▸ *c*), a nearly perfect reconstruction was obtained. Fig. 5 ▸(*d*) shows the r.m.s. variations of the amplitude and phase as a function of size. From the plot we infer for a particle with size below the extinction depth that the reconstruction is as good as that in the kinematical diffraction case. When the size approaches the *Pendellösung* distance, dynamical diffraction artefacts are dominant in the reconstruction and even the shape cannot be reconstructed correctly.

## 6. Dynamical diffraction from a strained nanoparticle   

**Figure 6:**
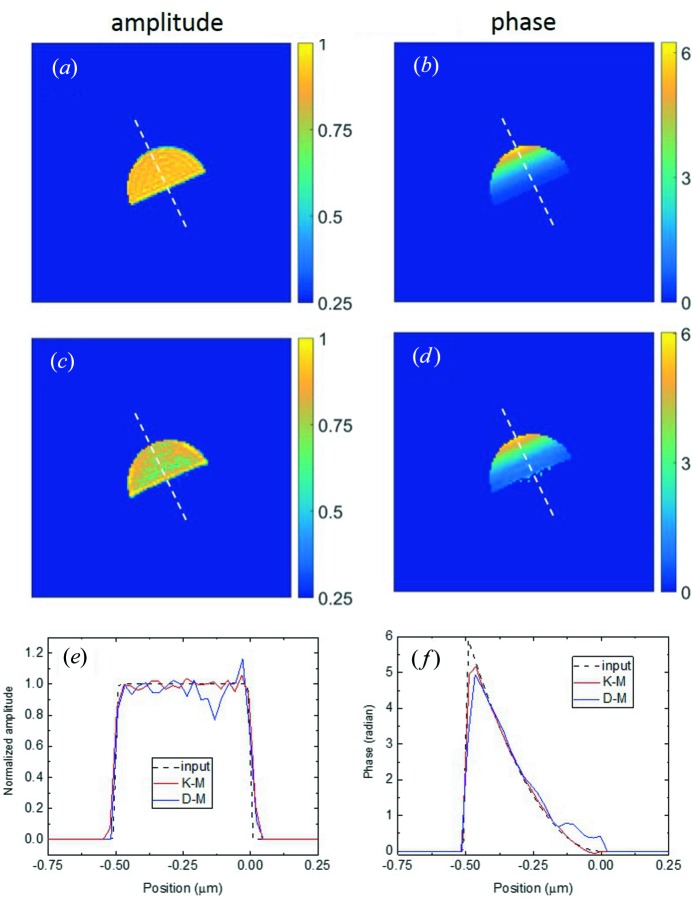
Reconstruction results for an Au hemisphere (500 nm radius) with a linear strain field. The Au 002 reflection at 7.5 keV is assumed. (*a*) and (*b*) are the cross-sectional views of the amplitude and phase obtained from the K-M data set, and (*c*) and (*d*) are from the D-M data set. Absorption and refraction corrections have been applied. (*e*) shows amplitude variations along the white dashed line in (*a*) and (*c*), compared with the input amplitude function (black). (*f*) shows phase variations along the white dashed line in (*b*) and (*d*), compared with the input phase function (black).

Another factor that affects the diffraction mode is the strain variation, which weakens dynamical diffraction effects (Yan *et al.*, 2007 ▸). Since all nanoparticles measured in a real experiment are imperfect and undergo some sort of deformation, it is important to understand how strongly dynamical diffraction will affect the reconstruction of a strained particle. In this section we perform a simulation on a hemisphere-shaped Au particle with a radius of 500 nm and a displacement field function along the reciprocal lattice vector direction only, 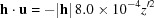 (see Fig. 3 ▸ *a*). This corresponds to a linear strain with a maximum value of 8 × 10^−4^ and a total phase variation of 2π. We chose the Au 002 reflection and an energy of 7.5 keV so that we could compare it with the case shown in Fig. 3 ▸. In Fig. 6 ▸ we depict reconstruction results based on data simulated using either the kinematical or the dynamical model. For clarity, absorption and refraction effects were removed from these images. Cross-sectional views of the amplitude and the phase of the reconstructed object function are depicted in Figs. 6 ▸(*a*) and 6 ▸(*b*) for the K-M data set and in Figs. 6 ▸(*c*) and 6 ▸(*d*) for the D-M data set. To make a quantitative comparison, a line was drawn across the particle, as shown in Figs. 6 ▸(*a*)–6 ▸(*d*), to plot profiles of the amplitude in Fig. 6 ▸(*e*) and the phase in Fig. 6 ▸(*f*). As evident from the plot, the K-M data set yields a fairly constant amplitude profile and a phase profile in good agreement with the input function. Some small deviations are observed. These are numerical errors due to the reconstruction algorithm itself. The reconstruction based on the D-M data set, however, produces a less uniform amplitude profile and a phase profile considerably different from the input phase function, particularly at the bottom of the particle. This observation suggests – for a large crystal with a constant structure factor – that if the reconstructed amplitude shows more fluctuation at the bottom this is a sign of dynamical diffraction artefacts.

In comparison to Figs. 3 ▸(*k*) and 3 ▸(*l*) where the crystal is perfect, we see much less pronounced dynamical diffraction artefacts in Figs. 6 ▸(*c*) and 6 ▸(*d*) in the presence of a strain variation, as expected. When the strain gradient increases further, we would expect a complete transition to the kinematical diffraction, but this discussion is out of the scope of this paper.

## 7. Conclusion   

In summary, we have performed a thorough theoretical investigation of reconstruction artefacts resulting from dynamical diffraction effects in Bragg coherent diffractive imaging. We showed that the kinematical diffraction model, the mathematical foundation of BCDI which can be formulated into a three-dimensional Fourier transform, is no longer valid for a large crystal, and the rigorous dynamical diffraction model has to be employed. The inconsistency between the forward dynamical diffraction formulism and the inverse BCDI reconstruction can lead to strong artefacts, which do not correspond to real structural variations inside the crystal. To illustrate this phenomenon, BCDI reconstructions were performed for Au hemispherical particles of various sizes. These reconstructions were based on synthetic diffraction data calculated using both kinematical and dynamical diffraction models. Even though these two models yield far-field diffraction patterns with subtle changes on the interference fringes, strong reconstruction artefacts arise in the latter case. In the case of dynamical diffraction, we showed that the root cause was the strong dependence of the transmitted wave on the rocking angle. An investigation of the size dependence of the dynamical artefacts in the reconstruction was carried out and showed that extinction depth and *Pendellösung* distance can serve as good criteria for predicting artefacts. Dynamical diffraction effects are negligible for particles with sizes below the extinction depth, while they can be dominant for particles with sizes approaching the *Pendellösung* distance. We also studied the dynamical diffraction effects from a hemispherical particle with a linear strain field, and we showed that dynamical diffraction artefacts were present, even though they were less pronounced than the effects observed in the unstrained case. From this study, we conclude that more caution should be exercised in the interpretation of BCDI reconstruction results for a nearly perfect crystal with a size larger than the extinction depth, and an appropriate combination of energy and reflection indices should be chosen to minimize dynamical artefacts in a real experiment whenever it is possible.
